# Role of NLRP3 Inflammasome in Lupus Nephritis and Therapeutic Targeting by Phytochemicals

**DOI:** 10.3389/fphar.2021.621300

**Published:** 2021-08-20

**Authors:** Dantong Wu, Lianjie Ai, Yanping Sun, Bingyou Yang, Sisi Chen, Qiuhong Wang, Haixue Kuang

**Affiliations:** ^1^ Key Laboratory of Chinese Materia Medica (Ministry of Education), Heilongjiang University of Chinese Medicine, Harbin, China; ^2^ Department of Laboratory Diagnostics, The First Affiliated Hospital, Heilongjiang University of Chinese Medicine, Harbin, China; ^3^ Department of General Surgery, The Second Affiliated Hospital of Harbin Medical University, Harbin, China; ^4^ Department of Rheumatology, The First Affiliated Hospital, Heilongjiang University of Chinese Medicine, Harbin, China; ^5^ Department of Natural Medicinal Chemistry, College of Pharmacy, Guangdong Pharmaceutical University, Guangzhou, China

**Keywords:** NLRP3 inflammasome, lupus nephritis, phytochemicals, podocyte, treatment

## Abstract

Systemic lupus erythematosus (SLE) is a multisystem autoimmune inflammatory condition that affects multiple organs and provokes extensive and severe clinical manifestations. Lupus nephritis (LN) is one of the main clinical manifestations of SLE. It refers to the deposition of immune complexes in the glomeruli, which cause kidney inflammation. Although LN seriously affects prognosis and represents a key factor of disability and death in SLE patients, its mechanism remains unclear. The NACHT, leucine-rich repeat (LRR), and pyrin (PYD) domains-containing protein 3 (NLRP3) inflammasome regulates IL-1β and IL-18 secretion and gasdermin D-mediated pyroptosis and plays a key role in innate immunity. There is increasing evidence that aberrant activation of the NLRP3 inflammasome and downstream inflammatory pathways play an important part in the pathogenesis of multiple autoimmune diseases, including LN. This review summarizes research progress on the elucidation of NLRP3 activation, regulation, and recent clinical trials and experimental studies implicating the NLRP3 inflammasome in the pathophysiology of LN. Current treatments fail to provide durable remission and provoke several sides effects, mainly due to their broad immunosuppressive effects. Therefore, the identification of a safe and effective therapeutic approach for LN is of great significance. Phytochemicals are found in many herbs, fruits, and vegetables and are secondary metabolites of plants. Evidence suggests that phytochemicals have broad biological activities and have good prospects in a variety of diseases, including LN. Therefore, this review reports on current research evaluating phytochemicals for targeting NLRP3 inflammasome pathways in LN therapy.

## Introduction

Systemic lupus erythematosus (SLE) is a prototypic multisystemic autoimmune condition with unclear etiology, attributed to loss of immune tolerance towards autoantigens and production of antinuclear autoantibodies (Bentham et al., 2015). Nuclear autoantigens/antibody complexes are deposited in multiple organs, resulting in tissue damage and severe clinical manifestations, including malar rash, arthralgia, fever, renal failure, and cardiovascular diseases (Tanaka et al., 2018; Durcan et al., 2019). Lupus nephritis (LN) is a main clinical manifestation of SLE; it negatively affects the quality of life of SLE patients and their long-term prognosis (Davidson, 2016; Fanouriakis et al., 2020). Approximately 50% of SLE patients develop renal disease at some stage (Almaani et al., 2017). Hence, it is necessary to further elucidate LN-related pathogenic mechanisms to develop practical therapeutic approaches.

Inflammasome refers to multiprotein immune complexes assembled by pattern recognition receptors (PRRs) in the cytoplasm. Its activation mediates inflammatory responses to cellular damage and pathogenic microbial infections (Franchi et al., 2012; Strowig et al., 2012). As an important component of the innate immunity, inflammasomes operate as a central pathogenic mechanism in various diseases (Duncan and Canna, 2018; Yang et al., 2019; Spel and Martinon, 2020). Several studies have reported that the NACHT, leucine-rich repeat (LRR), and pyrin (PYD) domains-containing protein 3 (NLRP3) inflammasome is involved in the occurrence and development of LN (Zhao et al., 2015; Zhang et al., 2018a; Lin Q. et al., 2019). This study summarizes current knowledge on the role of the NLRP3 inflammasome in the pathogenesis of LN.

Recently, phytochemicals have attracted much attention due to their cost, efficacy, and safety (Zhang et al., 2018b). We also present a review of several phytochemicals that have been shown to interfere with NLRP3 inflammasome-related signaling pathways in the context of LN.

## Overview of the NLRP3 Inflammasome

Inflammasomes mediate caspase-1 activation and induce the maturation and release of the proinflammatory cytokines IL-1 and IL-18, initiating a cascade of inflammatory responses. They also trigger caspase-1-dependent pyroptosis and induce cell death under pathologic inflammatory and stress conditions. Among different inflammasomes, the NLRP3 inflammasome has been the most extensively studied and elucidated (Hoffman et al., 2001). Different studies showed that activation of the NLRP3 inflammasome is closely related to multiple autoinflammatory diseases (Baroja-Mazo et al., 2014; Lu et al., 2017; Louvrier et al., 2020). Therefore, its role in LN pathogenesis has attracted more attention (Zhao et al., 2013a; Lech et al., 2015).

The NLRP3 inflammasome is a multiprotein oligomeric complex consisting of NLRP3, adapter apoptosis-associated speck-like (ASC) protein, and procaspase-1 (Martinon et al., 2009; Guo et al., 2015). NLRP3 inflammasome activation usually involves a priming step and an activation step (Latz et al., 2013). At priming, the engagement of PRRs, such as toll-like receptors (TLR) or cytokine receptors, activates the transcription factor NF-κB, which upregulates the expression of NLRP3 and pro-IL-1β and pro-IL-18 cytokine precursors (Mishra et al., 2013; Kelley et al., 2019).

At the activation stage, the NLRP3 inflammasome is activated through the recognition of various pathogen-associated molecular patterns (PAMPs) or damage-associated molecular patterns (DAMPs) molecules (Lamkanfi and Dixit, 2014; Swanson et al., 2019), including bacterial U1-snRNP, ATP, and dsDNA (Mariathasan et al., 2006; Shin et al., 2012; Muñoz-Planillo et al., 2013; Shin et al., 2013). These stimuli activate the NLRP3 inflammasome through several mechanisms that are not fully clarified. Current research suggests the potential involvement of K+ efflux, mitochondrial dysfunction, lysosomal rupture, or generation of reactive oxygen species (ROS) (Katsnelson et al., 2016; Yu and Lee, 2016; Han et al., 2018). In addition, NEK7 also interacts with NLRP3 and modulates the activation of NLRP3 inflammasomes (He et al., 2016; Chen et al., 2019).

Upon activation, the NLRP3 binds the stimulatory ligands and the NACHT domains promote self-mediated oligomerization of several NLRP3. NLRP3 combines with ASC through homotypic N-terminal PYD-PYD interactions. Assembled ASC recruits procaspase-1 through its C-terminal CARD-CARD interactions to form the NLRP3 inflammasome. Subsequently, the functional inflammasome initiates self-catalysis of procaspase-1 to caspase-1, followed by self-enzymatic hydrolysis of caspase-1, which generates the large p20 and small p10 subunits (Elliott and Sutterwala, 2015; Sharif et al., 2019). The p20 subunit converts pro-IL-1β and pro-IL-18 into IL-1β and IL-18, respectively, and promotes the secretion of these cytokines, which have a broad spectrum of inflammatory activities (Spalinger et al., 2017; Mende et al., 2018; Abbate et al., 2020). Simultaneously, activated caspase-1 cleaves gasdermin D (GSDMD) protein, leading to the formation of plasma membrane pores, triggering gasdermin D-mediated pyroptosis (Miao et al., 2010; He et al., 2015).

**FIGURE 1:**
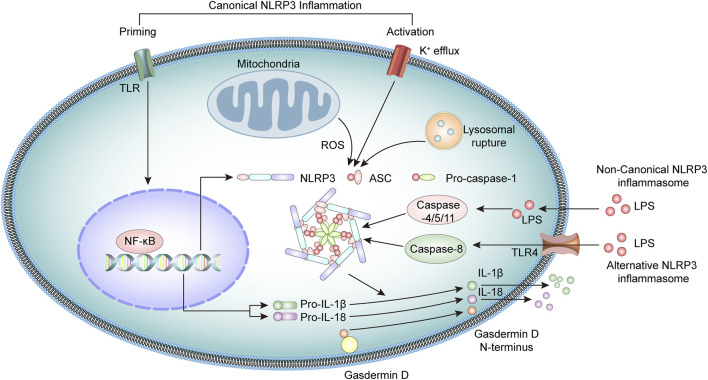
Overview of the molecular mechanisms of the NLRP3 inflammasome pathway.

Studies have identified a noncanonical inflammasome representing another crucial mechanism of inflammasome activation (Kayagaki et al., 2011). Noncanonical inflammasomes are generally activated by Gram-negative bacteria-derived molecules, including intracellular lipopolysaccharides and toxins. These inflammasomes mediate the activation of intracellular receptors caspase-11 in mice and caspase-4 and caspase-5 in humans, which are oligomerized after binding LPS and activate pyroptosis, together with inflammatory responses (Shi et al., 2014; Kayagaki et al., 2015; Lagrange et al., 2018). In addition, there is evidence that caspase-8 participates in the activation of NLRP3 inflammasome in human monocytes through an alternative NLRP3 inflammasome pathway (Xiang et al., 2020). Under LPS stimulation, the NLRP3 inflammasome activation is triggered by the TLR4-TRIF-RIPK1-FADD-CASP8 cascade signaling pathway, which then activates caspase-1 and secretes IL-1β (Gaidt et al., 2016; Zito et al., 2020) (Figure 1).

## Role of NLRP3 in LN

LN results from the interactions between genetics, epigenetics, sex hormones, environment, and other factors (Cunningham et al., 2019; Rahbar Saadat et al., 2019; Wardowska et al., 2019). The core pathogenesis of LN is a loss of self-tolerance, leading to accumulation or deposition of autoantibodies and immune complexes in the kidney, activating the complement system and causing chronic inflammation (Wada et al., 2019). Renal lesions in LN patients are characterized by glomerulonephritis, vascular injury, and tubular atrophy, which eventually may progress to renal fibrosis or even renal failure (Leatherwood et al., 2019).

Recent findings showed that NLRP3 and associated inflammatory cytokines, including IL-1β and IL-18, are elevated in the blood and nephritis biopsies from LN patients (da Cruz et al., 2020; Huang et al., 2020). Moreover, to systematically explore the mechanisms of NLRP3 on LN, different mouse models have been analyzed. In several models, NLRP3 and related components are increased in LN mice compared with controls (Honarpisheh et al., 2016; Bonomini et al., 2019). In MRL/lpr mice, a spontaneous lupus model, the expression of NLRP3, ASC, and active caspase l-p20 subunit protein was upregulated in the kidney, compared with control mice. Furthermore, the IL-1β level was upregulated in renal homogenates. Blocking upstream P2X7 receptor inhibited NLRP3 inflammasome assembly and reduced proteinuria (Zhao et al., 2013b). Intraperitoneal injection of pristane stimulates the body to produce autoantibodies, which has become a classic model for SLE (Freitas et al., 2017). In *Nlrp3* ^-R258W^ mutant mice, more severe renal pathological changes occurred when intraperitoneal injection with pristane and specific abrogation of *Nlrp3* ^-R258W^ expression in myeloid cells conferred a therapeutic benefit to lupus *Nlrp3* ^-R258W^ mutant (Lu et al., 2017).

Since the publication of histological classification criteria for LN, much attention has been focused on glomerulopathy in patients with LN and on the mechanisms of glomerular lesions. Subsequent revisions of previous knowledge have also been based on the pathological characteristics of glomerular damage (Azzouz et al., 2019; Dörner and Furie, 2019; Zhou et al., 2019). Endothelial cells, basement membrane, and podocytes form a glomerular filtration barrier, which plays a key role in maintaining the structure and function of the kidney (Nawata et al., 2018). Currently, research on NLRP3 in the context of LN mainly focuses on podocytes. The NLRP3 inflammasome activated in glomerular podocytes results in severe proteinuria in mouse lupus models and in patients with LN (Fu et al., 2017; Fu et al., 2019). Studies support a role for the NLRP3 inflammasome in promoting podocyte injury and proteinuria during LN. The level of activated caspase-1 in podocytes from LN NZM2328 mice with severe proteinuria is elevated, as well as in urine and tissue biopsies from patients with active LN. MCC950, an NLRP3 inhibitor, significantly inhibited caspase-1 in NZM2328 mouse podocytes by preventing NLRP3 inflammasome activation, ameliorated proteinuria, and reduced renal tissue damage. *In vitro*, sera from LN NZM2328 mice activated the NLRP3 inflammasome and increased the IL-1β level in podocytes by inducing ROS (Fu et al., 2017).

Evidence of RIP3 and necrotic pathway activity was found in podocytes from class IV LN patients and in the kidney of lupus-prone mice. GSK872, an inhibitor of RIP3, reduced anti-dsDNA antibody titer and the size and weight of the spleen, as well as RIP3 activation in podocytes. The upregulation of NLRP3, caspase-1 p20, and IL-1β levels induced by serum IgG from LN diseased NZM2328 mice could be inhibited by GSK872 (Guo et al., 2019).

As pointed out above, several studies have demonstrated that NLRP3 inflammasome components are involved in the pathogenesis of LN. Therefore, modulating NLRP3 inflammasome signals may represent a significant and promising target for LN management (Fu et al., 2019; Yang et al., 2020) (Table 1).

**TABLE 1:** Researches on NLRP3 inflammasome in lupus nephritis (LN).

Research type	Study subject	Mechanism	Ref.
Clinical research	LN patients	*NLRP3* rs10754558 was more frequent	da Cruz et al. (2020)
Clinical research	LN patients	Increased NLRP3 in tubular cells of LN class IV, positively correlated with the activity index (AI) score	Huang et al. (2020)
Experiment research	Pristane-induced female BALB/c mice	Upregulated NF-κB, iNOS, and NLRP3	Bonomini et al. (2019)
Experiment research	Podocytes of lupus-prone NZM2328 mice and LN patients	Activated NLRP3 inflammasome, caspase-1, and IL-1β	Fu et al. (2017)
Experiment research	Human podocytes	Enhanced NLRP3 inflammasome, caspase 1- p20, caspase 1, and IL-1β via stimulated with anti-dsDNA-positive serum	Fu et al. (2019)
Experiment research	Podocyte of lupus-prone NZM2328 mice	Detected interactions between RIP3 and NLRP3, upregulated NLRP3, and caspase-1 p20	Guo et al. (2019)
Experiment research	Female MRL/lpr mouse	NLRP3 was significantly high at 14 weeks	Honarpisheh et al. (2016)
Experiment research	Pristane-induced female Nlrp3^-R258W^ mice	Increased anti-dsDNA, total IgG, urine protein excretion, BUN, and urine creatinine	Lu et al. (2017)
Experiment research	Female NZB/W F1 mice	Upregulated ROS, NF-κB-p65,p- NF-κB-p65, NLRP3, caspase-1, and IL-1β	Yang et al. (2020)
Experiment research	Female MRL/lpr mouse	Increased P2X7, NLRP3, ASC, caspase-l p20, and IL-1β	Zhao et al. (2013a)
Experiment research	Female MRL/lpr mouse	Enhanced p-IκB, NF-κb-p65, NLRP3, ASC, and caspase-1 p20	Zhao et al. (2013b)

## Phytochemicals Targeting the NLRP3 Inflammasome in LN

**FIGURE 2:**
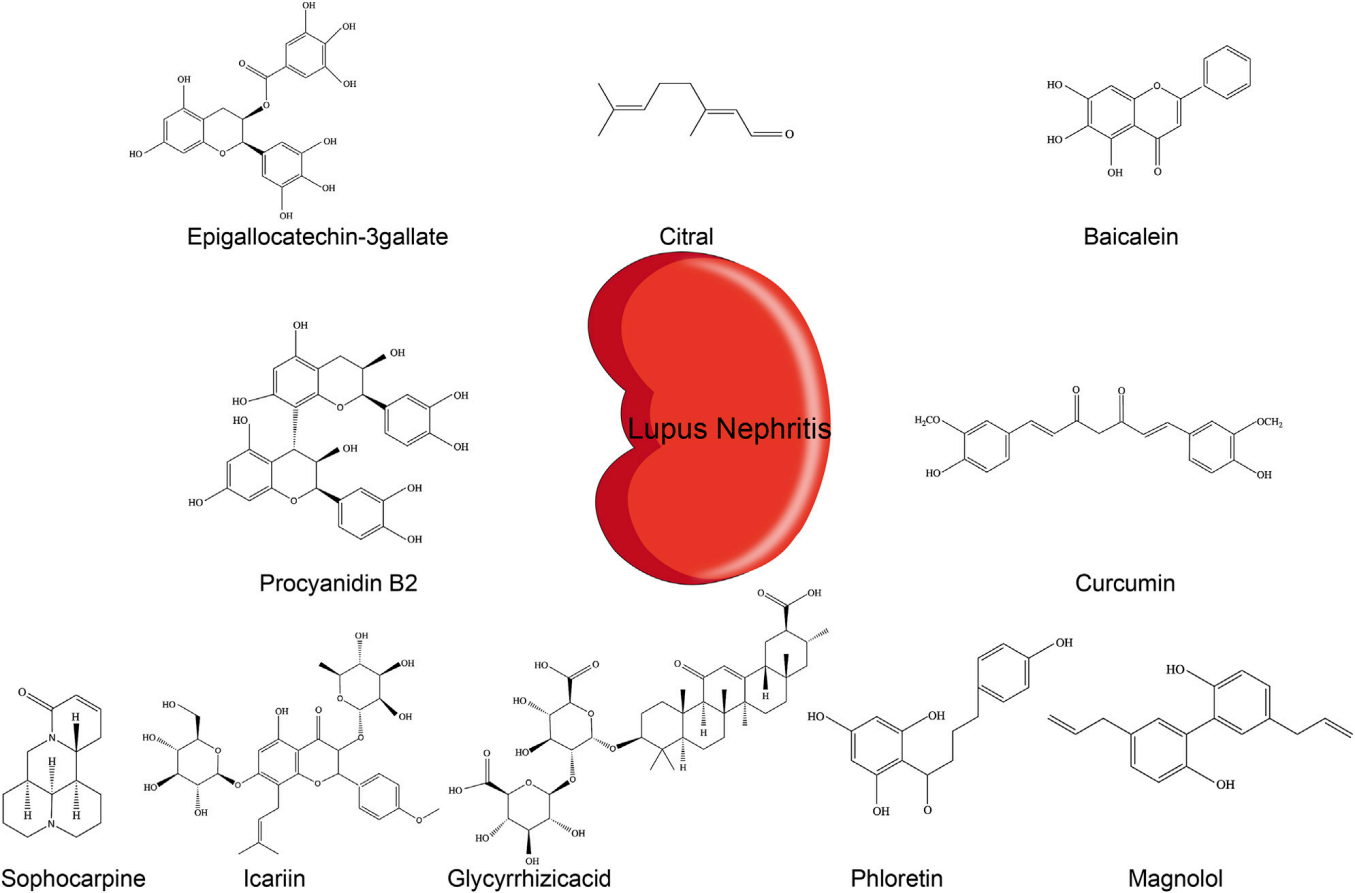
Chemical structures of different phytochemicals blocking NLRP3 inflammasome pathways in LN.

Common treatments for LN predominantly involve corticosteroids, antimalarial drugs, immunosuppressive agents, and biologics, which, although effective, are commonly associated with immunogenicity (Singh et al., 2016; Fava and Petri, 2019). Currently, available drugs do not meet the clinical demands for LN patients and are limited by suboptimal efficacy and severe side effects (Dall’Era et al., 2019; Murphy and Isenberg, 2019). There is increasing concern that drugs targeting the NLRP3 pathway may be appropriate for LN therapy (Lin T.-J. et al., 2019; Wu et al., 2020). Phytochemicals are secondary metabolites of plants with various bioactivity and are found in many herbs, fruits, and vegetables. Evidence suggests that phytochemicals have broad biological activities, including antioxidant, antiviral, and anti-inflammatory effects, and hold good prospects to treat autoimmune diseases and improve lipid metabolism among other diseases (Du et al., 2019; Guo et al., 2019; Hu et al., 2019a; McKee et al., 2020). To date, several phytochemicals have been shown to affect LN progression by inhibiting the NLRP3 inflammasome (Figure 2).

### Epigallocatechin-3-Gallate (EGCG)

EGCG is a bioactive polyphenol contained in green tea. EGCG has anti-inflammatory and antioxidant activities and represents a promising drug for the treatment of inflammatory diseases (Mi et al., 2017; Byun et al., 2018). EGCG reduced the ROS level in serum and urine of lupus-prone NZB/W F1 mice. This effect occurred likely through inhibition of the production of renal NAD(P)H oxidase, mediated by the nuclear factor E2-related factor 2 (Nrf2). This pathway could be the cause of the downregulation of NLRP3 mRNA and protein and subsequent attenuation of IL-1β and IL-18 secretion, resulting in lower proteinuria and improved renal functions (Tsai et al., 2011).

### Citral

Citral is a principal compound present in lemongrass and citrus fruits. It owes its therapeutic effects to its antioxidant (Li C.-C. et al., 2018) and antitumor activities Nigjeh et al., 2019). In a model of accelerated and severe LN (ASLN) in NZBxW F1 mice induced by lipopolysaccharide (LPS), citral treatment inhibited NLRP3 inflammasome signaling and IL-1β secretion by reducing ROS and COX-2. It also improved Nrf2 activation, ameliorated albuminuria secretion and renal function, and reduced the glomerulonephritis activity score and fibrinoid necrosis in the glomerulus. Consistent with these findings, citral inhibited caspase-1 activation and IL-1 secretion in ATP-induced macrophages *in vitro* (Ka et al., 2015).

### Baicalein

Baicalein is a flavonoid compound derived from the rhizome of the plant *Scutellaria baicalensis* Georgi. It has a broad spectrum of activities, including anticancer (Wu R. et al., 2018) and antioxidation (Woo et al., 2005). In a model of LN induced by pristane injections in BALB/C mice, baicalein treatment downregulated ROS production and enhanced Nrf2 activation. This effect was accompanied and prevented by NLRP3 inflammasome activation. Baicalein reduced albuminuria and improved renal function. Consistent with this effect, *in vitro* baicalein upregulated Nrf2 signaling and inhibited the NLRP3 inflammasome in LPS-primed myeloid-derived suppressor cells (MDSCs) (Li et al., 2019).

### Sophocarpine

Sophocarpine (SPC) is a natural quinolizidine alkaloid compound mainly found in the traditional Chinese herb *Sophorae flavescentis*. Numerous studies suggest that it exhibits various effects, including anti-inflammatory (Zou et al., 2019) and antitumoral (Zhang et al., 2016). Li et al. (2018) demonstrated that weekly gavage of MRL/lpr female mice suffering from LN with 100 mg/kg sophocarpine reduced the level of NLRP3 protein, ASC, caspase-1, and IL-1β in renal tissue, possibly through inhibition of the NF-κB activator IKKs. Treatment reduced serum and renal IL-1β, IL-6, and TNF-α. It also diminished proteinuria, reduced immune complex deposition in kidney, and significantly improved kidney function (Li et al., 2018a).

### Icariin

Icariin is a flavonol glucoside extracted from the herb Epimedium. Increasing evidence suggests that icariin possesses various pharmacological properties, such as antioxidant (Wu B. et al., 2018) and anti-inflammatory properties (El-Shitany and Eid, 2019).

In lupus-prone mice, treatment with 10 mg/kg/day of icariin for eight weeks reduced serum anti-dsDNA titer, decreased renal deposition of immune complexes, improved renal function, and alleviated the pathology. Moreover, icariin decreased IL-1β and TNF-α production in MRL/lpr mice, likely by inhibiting the NF-κB signaling pathway and the activation of NLRP3 and caspase-1 in kidney (Su B. et al., 2018).

### Glycyrrhizic Acid (GA)

GA is a natural extract of *Glycyrrhiza uralensis*. Clinical and experimental studies showed that it has many effects, including antioxidative (Umar et al., 2019) and immunoregulatory (Wu et al., 2016). In MRL/lpr mice, GA reduced serum uric acid and creatinine levels, thereby preventing severe renal injury. It exerted a protective activity by virtue of downregulating the NF-κB signaling pathway and reducing NLRP3 inflammasome activation. Assessment of proteins from the NF-κB and NLRP3 pathways by Western blot showed that GA inhibited the phosphorylation of NF-κB and IκBα and the activation of NLRP3, ASC, and caspase-1 in renal tissues and decreased serum and kidney IL-1β, IL-6, and TNF-α levels in treated MRL/lpr mice (Wang Y. Y.et al., 2017).

### Phloretin

Phloretin is a natural phenolic compound extracted from fruits. Recent studies revealed numerous significant activities of phloretin, including antioxidative, antiallergic (Huang et al., 2017a), and anti-inflammatory properties (Wu et al., 2019).

In MRL/lpr mice, phloretin attenuates renal injury and inhibits immune complex deposition (Hu and Yu, 2019b). Phloretin treatment decreased NLRP3, caspase-1, IL-1β, and TNF-α protein levels in renal tissue, as well as the levels of serum IL-1β and TNF-α. To further explore the regulation of NLRP3 by phloretin, Hu and colleagues assessed the NF-κB signaling pathway *in vitro*. Phloretin inhibited the cytoplasmic expression of *p*-IκB and p65 translocation to the nucleus and prevented NLRP3 inflammasome activation through inhibition of the NF-κB pathway.

### Magnolol (MG)

MG is a hydroxylated biphenyl compound extracted from *Magnolia officinalis*. Previous studies suggested that it has a variety of activities; it is used in treatment of melanoma (Emran et al., 2019) and anti-inflammatory (Liu et al., 2019).

In MRL/lpr mice, MG exhibited protective effects on glomerular and vascular lesions, decreased TNF-α in serum and renal tissues, and inhibited NLRP3 activation and IL-1β secretion through increased phosphorylation of IκB and level of IKK-α and NF-κB-p65 in kidney tissues (Huang et al., 2017b).

### Curcumin

Curcumin is a polyphenol extensively used in clinical treatment for cancer, bowel inflammation, and osteoarthritis (Marquardt et al., 2015; Bannuru et al., 2018; Burge et al., 2019). Curcumin can decrease proteinuria in female lupus-prone NZB/W F1 mice (Lee et al., 2013) and reduce PBMCs proliferation in patients with LN (Wang M. et al., 2017). In MRL/lpr mice, curcumin treatment downregulated serum anti-dsDNA antibody level, ameliorated proteinuria, reduced renal inflammation, and decreased caspase-1 p20 and renal IL-1β. In agreement with these findings, curcumin inhibited dsDNA-induced NLRP3 inflammasome activation in podocytes *in vitro* by reducing NLRP3 and caspase-1 p20 expression (Zhao et al., 2019).

### Procyanidin B2 (PCB2)

PCB2 is a bioactive phenolic compound isolated from apples, cocoa, and grapes. Previous research found that it holds diverse properties, such as modulation of the gut microbiota and anti-inflammatory and antioxidant effects (Su H. et al., 2018; Jiang et al., 2018; Endo et al., 2020). Recent evidence demonstrated that PCB2 directly represses the NLRP3 gene (He et al., 2018).

In MRL/lpr mice, treatment with PCB2 ameliorated LN renal lesions and decreased IL-18 and IL-1β levels in serum and renal tissues. PCB2 diminished anti-dsDNA antibody level and downregulated immune complex deposition in kidney. Moreover, He and colleagues also showed in MRL/lpr mice that silencing the NLRP3 gene reduced the production of IL-18 and IL-1β. A similar effect was found in PCB2 treated mice (He et al., 2018).

### Summary

ROS constitute a class of oxygen-containing compounds involved in cell metabolism (Reczek and Chandel, 2015; Lin et al., 2019; White et al., 2019). Under exogenous stimuli, such as silica and asbestos, ROS induces thioredoxin (TXN) dissociation from the thioredoxin-interacting protein (TXNIP). TXN binds and activates the NLRP3 inflammasome (Ding et al., 2018; Li et al., 2018b). Moreover, complexes deposited in the kidney activate the complement system and induce inflammatory cells that release proinflammatory factors and produce a large amount of ROS, inflammasome, thereby activating the NLRP3 inflammatory pathway and participate in the LN pathogenesis (Zhang et al., 2018). EGCG, citral, and baicalein downregulate NLRP3 levels by decreasing ROS, thereby reducing IL-1β and IL-18 secretion.

When externally stimulated, TLR signaling activates the transcription factor nuclear factor κB (NF-κB), upregulates NLRP3 expression, and promotes the production of the proinflammatory cytokines pro-IL-1β and pro-IL-18 in LN (Yi et al., 2017; Zhang et al., 2018). Sophocarpine, icariin, GA, phloretin, and magnolol could improve renal function by inhibiting the NF-kB pathway and reducing the expression of NLRP3. In addition, curcumin and PCB2 also could inhibit the expression of NLRP3 and have the potential to treat LN, according to the evaluation of anti-dsDNA antibody level and biochemical indexes (Table 2).

**TABLE 2:** Studies on phytochemicals inhibiting NLRP3 inflammasome activation in LN.

Molecular mechanisms	Phytochemicals	Category	Animal model	Dosage	Ref.
Suppression of ROS	Epigallocatechin-3-gallate	Polyphenol	Female NZB/W F1 mice	120 mg/kg for 22 weeks	Tsai et al. (2011)
	Citral	Monoterpenoid	Female NZB/W F1 mice	200 mg/kg for 5 weeks	Ka et al. (2015)
	Baicalein	Flavonoid	Pristane-induced female BALB/c mice	25 or 100 mg/kg for 8 weeks	Li et al. (2019)
Inhibition of NF-κB signaling pathway	Sophocarpine	Quinolizidine alkaloid	Female MRL/lpr mice	100 mg/kg for 18 weeks	Li et al. (2018a)
	Icariin	Flavonoid glucoside	Female MRL/lpr mice	10 mg/kg for 8 weeks	Su B. et al. (2018)
	Glycyrrhizic acid	Triterpene	Female MRL/lpr mice	20 or 40 mg/kg for 7 days	Wang Y. Y. et al. (2017)
	Phloretin	Phenolic	Female MRL/lpr mice	400 mg/kg for 8 weeks	Hu and Yu (2019b)
	Magnolol	Hydroxylated biphenyl	MRL/lpr mice	5 mg/kg for 8 weeks	Huang et al. (2017b)
Others	Curcumin	Polyphenol	Female MRL/lpr mice	200 mg/kg for 8 weeks	Zhao et al. (2019)
	Procyanidin B2	Phenolic	Female MRL/lpr mice	100 mg/kg for 8 weeks	He et al. (2018)

## Conclusion and Future Perspectives

SLE is an autoimmune disease involving multiple system damage. LN is an important renal complication of SLE, its clinical manifestations are complex and diverse, and the course of the disease is protracted and difficult to heal. If not treated in time, LN seriously affects the quality of life and survival rate of the patients. So far, the etiology and pathogenesis of LN remain unclear. As indicated above, activation of the NLRP3 inflammasome can promote the occurrence and development of the pathological processes leading to LN by causing inflammatory responses. With extensive research on inflammasomes, our understanding of its impact and mechanism of action on LN has become deeper and broader. Treatments targeting the NLRP3 inflammasome have attracted increasing attention (Li et al., 2020). Current studies on NLRP3 inflammasome in LN mainly focus on the canonical NLRP3 inflammasome pathway and lacks the detection of noncanonical NLRP3 inflammasome-related indicators, such as caspase-11, caspase-4, and caspase-5. Compared with the inactive LN subgroup and healthy controls, the serum level of caspase-8 increased significantly in active LN (Petrackova et al., 2017). It is worth further verification whether caspase-8 is involved in the pathogenesis of LN through an alternative NLRP3 inflammasome pathway. The research on the noncanonical NLRP3 inflammasome and alternative NLRP3 inflammasome pathway is still in infancy, but whether they are involved in LN and what role they play in it deserve our attention and exploration.

At present, treatment options for LN are limited. The main treatments consist of corticosteroids, antimalarials, and immunosuppressants, but these induce adverse reactions, such as immunosuppression and increased infection susceptibility. Therefore, it is extremely valuable to develop more effective treatments involving drugs with safety. This article summarizes the research progress made in recent years related to the therapeutic effects of phytochemicals on LN, acting through the NLRP3 inflammasome (Figure 2). The research of phytochemicals on NLRP3 in LN is limited to NLRP3 itself and the inflammatory factors IL-1β and pro-IL-18. In the context of lupus, there may be more specific targets upstream or downstream of NRLP3. This could help identify the role of phytochemicals on these specific molecules in other types of pathology and be tested in the context of lupus. A better understanding of the activation mechanisms of the NLRP3 inflammasome in LN will provide new ideas and approaches for the treatment of LN by phytochemicals. In addition, the clinical treatment of LN requires long-term medication, and the safety evaluation of the therapeutic dose and duration of phytochemicals needs to be further verified. This is a promising area, but there are many gaps that we need to fill in. There are significant differences in the incidence and severity of LN between different regions and races globally, and women are higher than men (Almaani et al., 2017). As summarized in this article, most of the studies on NLRP3 inflammasome in LN reported so far have been conducted in mouse models, and studies on LN patients with different genetic backgrounds will further determine the role of NLRP3 inflammasome.
